# Circuit dynamics of the olfactory pathway during olfactory learning

**DOI:** 10.3389/fncir.2024.1437575

**Published:** 2024-07-05

**Authors:** Yutian J. Zhang, Jason Y. Lee, Kei M. Igarashi

**Affiliations:** ^1^Department of Anatomy and Neurobiology, School of Medicine, University of California, Irvine, Irvine, United States; ^2^Department of Biomedical Engineering, Samueli School of Engineering, University of California, Irvine, Irvine, United States; ^3^Center for Neural Circuit Mapping, School of Medicine, University of California, Irvine, Irvine, United States; ^4^Center for the Neurobiology of Learning and Memory, University of California, Irvine, Irvine, United States; ^5^Institute for Memory Impairments and Neurological Disorders, University of California, Irvine, Irvine, United States

**Keywords:** olfactory, lateral entorhinal cortex (LEC), Olfactory learning, olfactory cortex, hippocampus

## Abstract

The olfactory system plays crucial roles in perceiving and interacting with their surroundings. Previous studies have deciphered basic odor perceptions, but how information processing in the olfactory system is associated with learning and memory is poorly understood. In this review, we summarize recent studies on the anatomy and functional dynamics of the mouse olfactory learning pathway, focusing on how neuronal circuits in the olfactory bulb (OB) and olfactory cortical areas integrate odor information in learning. We also highlight in vivo evidence for the role of the lateral entorhinal cortex (LEC) in olfactory learning. Altogether, these studies demonstrate that brain regions throughout the olfactory system are critically involved in forming and representing learned knowledge. The role of olfactory areas in learning and memory, and their susceptibility to dysfunction in neurodegenerative diseases, necessitate further research.

## 1 Introduction

Olfaction is a crucial ability for animals to detect environmental cues that are relevant for survival such as rewarding foods or dangerous predators. The sense of smell is also critical for human beings when we are involved in daily activities and experience the surrounding world. In the recent COVID-19 pandemic, nearly 88% of patients experienced olfaction loss in the short term (Lechien et al., 2020). A study also observed long-term structural changes in the brain such as tissue damage in the primary olfactory cortex and limbic regions that are functionally connected with the olfactory pathway (Douaud et al., 2022). Structural changes were also observed in memory-related regions including the entorhinal cortex and the hippocampus (Douaud et al., 2022). Furthermore, olfactory loss is associated with cognitive decline and declarative memory impairment in long-term COVID-19 patients (Fiorentino et al., 2022). Due to the overlapping vulnerability of olfactory and memory-related regions, it is even possible that long-term Covid infection could contribute to increased risks of neurodegenerative diseases like Alzheimer’s disease (AD). Thus, it is increasingly urgent to investigate the neural circuit dynamics behind olfaction and memory. Among different model organisms, rodents are particularly adept in olfactory tasks and possess multiple homologies with higher mammals, offering valuable insights into the neural circuits and dynamic representations of olfaction (Ache and Young, 2005). As previous research has established the basic odor representation component of the olfactory system, the current field has introduced new perspectives on how animals associate odor cues with specific outcomes, and how the neural representations of odors change across learning along different regions of the olfactory pathway. In this review, we will cover the circuit mechanisms of olfactory regions and their dynamics during olfactory learning.

### 1.1 Anatomy of olfactory pathway in rodents

**FIGURE 1:**
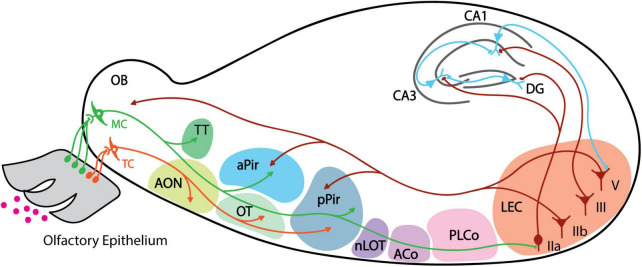
Schematic of mouse olfactory pathway anatomy. Individual areas are highlighted with different colors. Projections from mitral cell are shown in green, tufted cell projections in orange, back projection from LEC layers IIb and V in dark red, and projections from hippocampus neurons in blue. Arrows indicate axonal projections, and boutons indicate direct synaptic connection. MC, mitral cell; TC, tufted cell; AON, anterior olfactory nucleus; TT, tenia tecta; OT, olfactory tubercle; aPir, anterior piriform cortex; pPir, posterior piriform cortex; nLOT, nucleus of the lateral olfactory tract; ACo, anterior cortical amygdaloid; PLCo, posterolateral cortical amygdaloid nucleus; LEC, lateral entorhinal cortex; DG, dentate gyrus.

Olfactory information is first detected by olfactory sensory neurons (OSNs) located in the olfactory epithelium (OE) within the nasal cavity (Buck, 1996; Mori et al., 1999) (Figure 1). OSNs relay signals to the olfactory bulb (OB), which contains two types of projection neurons: mitral cells (MCs) and tufted cells (TCs) (Shepherd, 2004). From there, MCs and TCs send information to several olfactory cortical areas in the brain (Miyamichi et al., 2011; Igarashi et al., 2012; Nagayama et al., 2014). Olfactory cortex is defined as areas that receive direct input from the OB, including 9 major brain regions: anterior olfactory nucleus (AON), olfactory tubercule (OT), anterior piriform cortex (aPir), posterior piriform cortex (pPir), lateral entorhinal cortex (LEC), nucleus of the lateral olfactory tract (nLOT), anterior cortical amygdaloid nucleus (ACo), posterolateral cortical amygdaloid nucleus (PLCo) and tenia tecta (TT) (Paxinos, 2004; Shepherd, 2004; Igarashi et al., 2012). TCs signal mainly to the anterior portion of the olfactory cortex including the AON and the OT, while MCs are thought to be the main output neurons of OB and project to all the olfactory cortical regions (Igarashi et al., 2012; Nagayama et al., 2014; Chen et al., 2022) (Figure 1).

The organization of the olfactory system is unique. First, the olfactory system does not have a thalamic relay and contains only three primary layers of information processing: OE as the first layer, OB as the second, and the remaining olfactory cortical regions tied for the third. It is surprising that olfaction could be encoded in this three-layer hierarchy system as this is not observed in other sensory modalities such as the visual system where the primary (V1), secondary (V2), and third (V4) visual cortex are sequentially connected. The second unique feature is the odor representation pattern in the olfactory cortex. While the odor representation of OB neurons is spatially organized (Mori et al., 2006), the piriform cortex (PC), which has the largest area among the olfactory cortex, receives divergent axonal projections from mitral cells (Miyamichi et al., 2011; Igarashi et al., 2012) and responds to odors in distributed neuronal ensembles lacking a topographical pattern (Stettler and Axel, 2009). This is also not observed in other associational sensory cortical regions like V1 or primary auditory cortex in mammals (Malach, 1989; Ojima et al., 1991) but only in higher regions such as V4 and primary somatosensory cortex (area 3b) in monkeys (Hansen et al., 2007), suggesting the role of olfactory cortex as a higher association cortex.

### 1.2 The role of olfactory regions in learning

Previous studies have identified the anatomical connections of the olfactory bulb and olfactory cortex regions, but the functions of these regions remain largely unknown. By contrast, as most prior studies measured the neuronal responses of OB in basic odor detection, studies from the past few years have begun to reveal the function of OB in learning. Intrinsic optical imaging of OB showed an increase in the number of activated glomeruli in mice trained on a go/no go odor discrimination task, and this learning-induced potentiation lasted up to 5 weeks (Abraham et al., 2014). Targeting recordings specifically to MCs showed that, after learning, synchronized activity in MCs carries information about odor value (rewarded or unrewarded) (Doucette et al., 2011). Additionally, when task difficulties depended on the similarities of odorants that mice need to discriminate, the fraction of responsive MCs increased over weeks for mice trained with the difficult task, and decreased in mice with the easy task, demonstrating experience-dependent plasticity in the OB (Chu et al., 2016). Moreover, recordings from evoked field postsynaptic potentials demonstrated that connections between OB-PC inputs were enhanced during rule learning (Cohen et al., 2015). These studies suggest that the OB not only functions as a sensory relay station but also endures plasticity-related changes across learning.

Recent studies have also found plasticity-related changes in some of the olfactory cortex regions, including the OT, AON, TT and the LEC. A paper reported learning-dependent plasticity in OT, with the anteromedial domain of OT responding more to appetitive odor cues and the lateral domain responding to aversive cues after learning (Murata et al., 2015). This suggests that OT is involved in the expected outcome representation of specific stimuli, with activation of different domains based on the outcome valence. Moreover, optogenetic stimulation of either the OB-OT or Pir-OT pathway alone elicited food-searching behavior in mice trained with reward, as well as shock avoidance in aversively-trained mice (Sha et al., 2023). Both of these synaptic connections could potentially explain learning-induced potentiation of the OT (Sha et al., 2023). Additionally, TT neurons are tuned to specific task elements with learning, such as temporal epochs and approach behavior (Shiotani et al., 2020). For AON, memory engram-like activities were detected using cFos labeling after mice were exposed to an odor-context paradigm, and inhibition of AON engram activity disrupted the odor-contextual associative memory recall (Aqrabawi and Kim, 2020). Anatomically, topographical projections from the hippocampus to AON were revealed previously (Aqrabawi and Kim, 2018b), and this HPC-AON input is necessary for forming olfactory-contextual memory (Aqrabawi and Kim, 2018a). Collectively, these experiments provide new insights into how we understand the olfactory system – that is, that each of the olfactory regions is presumably involved in specific aspects of olfactory learning. Future studies should further clarify the distinct roles of individual regions and identify the circuit mechanisms supporting learning-induced changes in these olfactory areas.

## 2 The role of the LEC in olfactory learning

### 2.1 Anatomical connections of LEC

**FIGURE 2:**
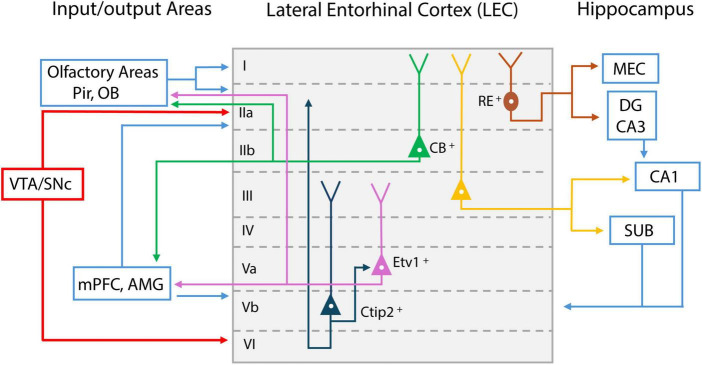
Schematic representation of lateral entorhinal cortex (LEC) layer input/output projections. Inputs from olfactory bulb (OB) and piriform cortex (Pir) reach LEC layer I; Reelin (RE) positive cells project to medial entorhinal cortex (MEC) and dentate gyrus (DG) (shown in dark red); Calbindin (CB) positive pyramidal cells in layer II project to olfactory areas, medial prefrontal cortex (mPFC), and amygdala (AMG) (shown in green); Layer III pyramidal cells mainly project to CA1 and subiculum (SUB) (shown in yellow). Layer Va cells express E twenty-six variant transcription factor 1 (Etv1), and project to mPFC and AMG, as well as olfactory areas (shown in purple); Feedback from these areas reach layer Vb. Layer Vb cells express Chicken ovalbumin upstream promoter transcription factor-interacting protein 2 (Ctip2) and project within the LEC (shown in dark blue). LEC layer II and VI receive dopaminergic inputs from midbrain ventral tegmental area (VTA) and substantial nigra pars compacts (SNc) (shown in red).

Among the olfactory cortical areas, LEC is unique in that it is also a constituent of the memory system. LEC is situated between olfactory regions and the hippocampus, serving as an information transfer station between the two. LEC is a six-layer structure with distinct input/output properties and distinct cell types comprising each layer (Figure 2). It receives direct inputs from MCs via the LOT and makes bidirectional connections with Pir (Agster and Burwell, 2009; Igarashi et al., 2012; Diodato et al., 2016). LEC also receives inputs from the insular cortex (Agster and Burwell, 2009). These axon terminals reach LEC mainly in the superficial layer I, which contains mostly the apical dendrites of layer II cells (Burwell and Amaral, 1998). Inputs from the perirhinal and postrhinal cortex terminate at layer II, which may allow for the integration of object representations with olfactory cues (Doan et al., 2019) (Figure 2).

LEC layer II is a dense cell layer. Layer IIa contains mostly reelin^++^ (RE^+^) principal cells (fan cells) and IIb contains calbindin^+^ (CB^+^) pyramidal cells. Fan cells project mainly via the perforant pathway to the dentate gyrus (DG), where information is sent to hippocampal CA3 and CA1 for further processing (Leitner et al., 2016) (Figure 2). This allows LEC to serve as the major gateway for sensory information entering memory processing regions. Fan cells that project to DG also project to the superficial layer of the medial entorhinal cortex (MEC), allowing feedforward inhibition to pyramidal cells in MEC circuits (Vandrey et al., 2022). LEC layer IIb CB^+^ pyramidal cells do not innervate DG but rather send feedback projections mainly to the OB, Pir, contralateral LEC, and neocortical areas such as the medial prefrontal cortex (mPFC) (Kerr et al., 2007; Agster and Burwell, 2009; Leitner et al., 2016; Ährlund-Richter et al., 2019).

LEC layer III contains pyramidal cells projecting mostly to CA1 and subiculum (SUB), and projections from these hippocampal regions terminate back in the deep layer of LEC. In deep layer V, neurons project to the mPFC, amygdala (AMG), olfactory bulb, anterior olfactory nucleus, and piriform cortex (de Olmos et al., 1978; Insausti et al., 1997). Recently, layer V cells have been divided into layers Va and Vb, using two marker proteins. Layer Va cells express E twenty-six (ETS) variant transcription factor 1 (Etv1), whreas Layer Vb cells express chicken ovalbumin upstream promoter transcription factor (COUP-TF) interacting protein 2 (Ctip2) (Sürmeli et al., 2016; Ohara et al., 2018) (Figure 2). Layer Va cells are the main output neurons projecting outside the LEC, whereas layer Vb cells are considered mostly for intrinsic projections within the LEC, synapsing onto both layer Va and the superficial layer of LEC (Ohara et al., 2018). Based on the looping structure of input/output connectivity in LEC sublayers (superficial layer LEC → hippocampus → deep layer LEC → neocortex), it is likely that the deep layer LEC receives olfactory-memory representations from the hippocampus and sends this feedback information to various cortical regions, where it is integrated for higher-level cognition and long-term memory maintenance.

### 2.2 LEC involvement in learning

Due to its intricate input/output connection with the hippocampus, multiple lines of research have focused on LEC’s role in learning and memory. While the MEC was thought to represent the spatial component of learning (“where”), as supported by the discoveries of spatially modulated grid cells (Fyhn et al., 2004; Hafting et al., 2005), LEC cells have low spatially selective firing (Hargreaves et al., 2005). Rather, LEC may represent visual, olfactory, and somatosensory information about items and objects (Young et al., 1997; Deshmukh and Knierim, 2011; Tsao et al., 2013; Igarashi et al., 2014). To identify how LEC neurons encode memories of items/objects, we recently recorded layer IIa fan cells using optogenetic assisted cell-type-specific recording method when mice were learning odor-outcome association (Lee et al., 2021). We found a group of LEC fan cells that developed spike responses to both newly learned rewarded odor cues and pre-learned rewarded cues, a signature of generalization during learning. Another group of fan cells responded only to punished cues. These results suggest that the LEC forms a “cognitive map” of odor items during memory encoding. The cognitive map refers to an internal brain representation of a physical spatial map, first conceptualized by Tolman from his observations on rats running in a maze (Tolman, 1948), then supported by the discovery of place cells in the hippocampus (O’Keefe and Dostrovsky, 1971). Recently, the concept of the cognitive map has been extended to non-spatial elements (Behrens et al., 2018). The Lee et al. work suggested that the LEC classifies and stores information of items depending on their associated reward or punishment outcomes, a signature of cognitive map formation about learned items (Lee et al., 2021; Igarashi et al., 2022). We consider that the concept of cognitive map is similar to the idea of “memory schema,” a term for “acquired knowledge” in psychological studies (Bartlett, 1932; Craik, 1943; Tse et al., 2007). In this context, the LEC (and presumably other brain regions) formulate schema from previous learning, and use schema to guide the acquisition of subsequently learned knowledge, which is referred to as “assimilation” (Igarashi et al., 2022).

## 3 Neuromodulatory inputs underlying olfactory learning

The olfactory areas receive dense neuromodulatory inputs presumably contributing to olfactory learning, including dopaminergic projections from the midbrain ventral tegmental area (VTA) and substantia nigra (SN), cholinergic inputs from the diagonal band of Broca (DB) and the basal forebrain, and serotonergic inputs from the raphe nuclei (Hokfelt et al., 1974, Björklund and Dunnett, 2007; Petzold et al., 2009). Among these neuromodulatory systems, the most extensive research has been conducted on dopamine (DA). Even though OB does not receive direct DA inputs from midbrain VTA, it has been indicated that the OB contains numerous dopaminergic neurons within the glomerular layer (Halasz et al., 1981) and expresses DA type 2 receptor (D2R) (Coronas et al., 1997; Koster et al., 1999). OT, as part of the ventral striatal system, receives extensive DA inputs from VTA (Ikemoto, 2007; Zhang et al., 2017), and phase stimulation of the DA terminals in OT induces neuronal plasticity of cue-reward pairing (Oettl et al., 2020). Moreover, LEC receives dense DA inputs from the VTA/SN, found mainly in layers II and VI (Hokfelt et al., 1974; Fallon et al., 1978; Björklund and Dunnett, 2007) (Figure 2). However, whether DA inhibits or facilitates LEC layer II synaptic transmission remains controversial (Caruana and Chapman, 2008; Glovaci and Chapman, 2015). We recently demonstrated that DA inputs from the VTA control the development of cue-reward representation in LEC layer IIa fan cells during associative learning (Lee et al., 2021). Additionally, both OB and Pir receive cholinergic inputs from the horizontal limbs of diagonal band of Broca (HDB), suggesting that acetylcholine could also modulate these regions in learning (Luskin and Price, 1982; Zaborszky et al., 1986). For example, while previous exposure to similar odorants increases the odor discrimination ability, this learned enhancement was reversed by an acetylcholine antagonist (Fletcher and Wilson, 2002). These results collectively indicate that neuromodulatory inputs such as DA and acetylcholine could guide olfactory memory formation and control information flow in the entorhinal-hippocampal circuit for learning.

## 4 Discussion and conclusion

In this review, we attempted to summarize currently available knowledge about the mouse olfactory system in learning with anatomical underpinnings. The fact that neocortical regions like the medial prefrontal cortex (mPFC) receive direct input from several olfactory cortical regions (i.e. PC, TT, AON, LEC) suggests that olfactory information is directly integrated for cognitive functions such as decision-making and adaptive responses (Witter et al., 2000; Agster and Burwell, 2009; DeNardo et al., 2015; Diodato et al., 2016; Moberly et al., 2018; Loureiro et al., 2019). Also of note is the strong connectivity between olfactory areas and the amygdala (Price, 2003), which could provide a neural basis for the emotional aspect of olfactory memory.

We also emphasized the special role of LEC as an interface for the olfactory memory circuit in associative learning and its modulation by dopaminergic inputs. The LEC forms cognitive maps for non-spatial olfactory items during olfactory learning (Igarashi et al., 2022). It remains largely unknown whether the function of cognitive map/schema formation, found in the LEC, is shared in other olfactory regions, or their target cortical areas. Future work is needed to decipher their roles in cognitive map formation during learning. Another important topic for future study is the critical role of the LEC in AD. Olfactory impairment is demonstrably correlated with AD and is amongst the first symptoms reported by many AD patients (Waldton, 1974; Serby et al., 1991). Moreover, the LEC is thought to be one of the first regions exhibiting histological and functional signatures of AD (Van Hoesen et al., 1991; Braak and Braak, 1992; Igarashi, 2023). It is likely that LEC dysfunction underlies the progression of both smell and memory loss. How the early stage of olfactory representations is altered in disease and affects higher-level cognition requires future investigation.

Our experience with the surrounding environment involves a convergence of many sensory modalities. An interesting property of olfactory cortical regions (e.g., OT, Pir and LEC) is their multi-modal representations outside of olfaction. Pir receives projections from the primary auditory cortex (A1) (Budinger et al., 2006), and studies have shown that OT cells respond to auditory stimuli (Wesson and Wilson, 2010). In LEC, somatosensory and visual inputs may converge with olfactory information to form a more complete representation of the outside world (Hoogland et al., 1987; Burwell and Amaral, 1998). The mechanisms of multimodal features of olfactory regions also call for investigation in future studies.

## Author contributions

YZ: Writing – review and editing, Writing – original draft. JL: Writing – review – editing, Writing – original draft. KI: Writing – review – editing, Writing – original draft.

## References

[B1] Abraham N. M. Vincis R. Lagier S. Rodriguez I. Carleton A. (2014). Long term functional plasticity of sensory inputs mediated by olfactory learning. *Elife* 3:e02109. 10.7554/eLife.02109 PMC3953949 24642413

[B2] Ache B. W. Young J. M. (2005). Olfaction: Diverse species, conserved principles. *Neuron* 48 417–430. 16269360 10.1016/j.neuron.2005.10.022

[B3] Agster K. L. Burwell R. D. (2009). Cortical efferents of the perirhinal, postrhinal, and entorhinal cortices of the rat. *Hippocampus* 19 1159–1186. 19360714 10.1002/hipo.20578 PMC3066185

[B4] Ährlund-Richter S. Xuan Y. Van Lunteren J. A. Kim H. Ortiz C. Dorocic I. P. (2019). A whole-brain atlas of monosynaptic input targeting four different cell types in the medial prefrontal cortex of the mouse. *Nat. Neurosci.* 22:657. 10.1038/s41593-019-0354-y 30886408

[B5] Aqrabawi A. J. Kim J. C. (2018b). Topographic organization of hippocampal inputs to the anterior olfactory nucleus. *Front. Neuroanat.* 12:12. 10.3389/fnana.2018.00012 29520221 PMC5827092

[B6] Aqrabawi A. J. Kim J. C. (2018a). Hippocampal projections to the anterior olfactory nucleus differentially convey spatiotemporal information during episodic odour memory. *Nat. Commun.* 9:2735. 10.1038/s41467-018-05131-6 30013078 PMC6048034

[B7] Aqrabawi A. J. Kim J. C. (2020). Olfactory memory representations are stored in the anterior olfactory nucleus. *Nat. Commun.* 11:1246. 10.1038/s41467-020-15032-2 PMC7060254 32144256

[B8] Bartlett F. C. (1932). *Remembering. A study in experimental and social psychology.* Cambridge: Cambridge University Press.

[B9] Behrens T. E. J. Muller T. H. Whittington J. C. R. Mark S. Baram A. B. Stachenfeld K. L. (2018). What is a cognitive map? Organizing knowledge for flexible behavior. *Neuron* 100 490–509. 30359611 10.1016/j.neuron.2018.10.002

[B10] Björklund A. Dunnett S. B. (2007). Dopamine neuron systems in the brain: An update. *Trends Neurosci.* 30 194–202. 10.1016/j.tins.2007.03.006 17408759

[B11] Braak H. Braak E. (1992). The human entorhinal cortex: Normal morphology and lamina-specific pathology in various diseases. *Neurosci. Res.* 15 6–31. 1336586 10.1016/0168-0102(92)90014-4

[B12] Buck L. B. (1996). Information coding in the vertebrate olfactory system. *Annu. Rev. Neurosci.* 19 517–544. 8833453 10.1146/annurev.ne.19.030196.002505

[B13] Budinger E. Heil P. Hess A. Scheich H. (2006). Multisensory processing via early cortical stages: Connections of the primary auditory cortical field with other sensory systems. *Neuroscience* 143 1065–1083. 17027173 10.1016/j.neuroscience.2006.08.035

[B14] Burwell R. D. Amaral D. G. (1998). Cortical afferents of the perirhinal, postrhinal, and entorhinal cortices of the rat. *J. Comp. Neurol.* 398 179–205. 9700566 10.1002/(sici)1096-9861(19980824)398:2<179::aid-cne3>3.0.co;2-y

[B15] Caruana D. A. Chapman C. A. (2008). Dopaminergic suppression of synaptic transmission in the lateral entorhinal cortex. *Neural Plast.* 2008:203514. 10.1155/2008/203514 PMC2519792 18769495

[B16] Chen Y. Chen X. Baserdem B. Zhan H. Li Y. Davis M. B. (2022). High-throughput sequencing of single neuron projections reveals spatial organization in the olfactory cortex. *Cell* 185:4117–4134.e28. 10.1016/j.cell.2022.09.038 36306734 PMC9681627

[B17] Chu M. W. Li W. L. Komiyama T. (2016). Balancing the robustness and efficiency of odor representations during learning. *Neuron* 92 174–186. 10.1016/j.neuron.2016.09.004 27667005 PMC5061050

[B18] Cohen Y. Wilson D. A. Barkai E. (2015). Differential modifications of synaptic weights during odor rule learning: Dynamics of interaction between the piriform cortex with lower and higher brain areas. *Cereb. Cortex* 25 180–191. 10.1093/cercor/bht215 23960200 PMC4415065

[B19] Coronas V. Srivastava L. K. Liang J. J. Jourdan F. Moyse E. (1997). Identification and localization of dopamine receptor subtypes in rat olfactory mucosa and bulb: A combined in situ hybridization and ligand binding radioautographic approach. *J. Chem. Neuroanat.* 12 243–257. 10.1016/s0891-0618(97)00215-9 9243344

[B20] Craik K. J. W. (1943). *The nature of explanation.* Cambridge: Cambridge University Press.

[B21] de Olmos J. Hardy H. Heimer L. (1978). The afferent connections of the main and the accessory olfactory bulb formations in the rat: An experimental Hrp-study. *J. Comp. Neurol.* 181 213–244. 10.1002/cne.901810202 690266

[B22] DeNardo L. A. Berns D. S. Deloach K. Luo L. (2015). Connectivity of mouse somatosensory and prefrontal cortex examined with trans-synaptic tracing. *Nat. Neurosci.* 18 1687–1697. 10.1038/nn.4131 26457553 PMC4624522

[B23] Deshmukh S. S. Knierim J. J. (2011). Representation of non-spatial and spatial information in the lateral entorhinal cortex. *Front. Behav. Neurosci.* 5:69. 10.3389/fnbeh.2011.00069 22065409 PMC3203372

[B24] Diodato A. De Brimont M. R. Yim Y. S. Derian N. Perrin S. Pouch J. (2016). Molecular signatures of neural connectivity in the olfactory cortex. *Nat. Commun.* 7:12238. 10.1038/ncomms12238 PMC4960301 27426965

[B25] Doan T. P. Lagartos-Donate M. J. Nilssen E. S. Ohara S. Witter M. P. (2019). Convergent projections from perirhinal and postrhinal cortices suggest a multisensory nature of lateral, but not medial, entorhinal cortex. *Cell Rep.* 29:617–627.e7. 10.1016/j.celrep.2019.09.005 31618631

[B26] Douaud G. Lee S. Alfaro-Almagro F. Arthofer C. Wang C. Y. Mccarthy P. (2022). SARS-CoV-2 is associated with changes in brain structure in Uk Biobank. *Nature* 604:697. 10.1038/s41586-022-04569-5 PMC9046077 35255491

[B27] Doucette W. Gire D. H. Whitesell J. Carmean V. Lucero M. T. Restrepo D. (2011). Associative cortex features in the first olfactory brain relay station. *Neuron* 69 1176–1187. 10.1016/j.neuron.2011.02.024 21435561 PMC3064824

[B28] Fallon J. H. Koziell D. A. Moore R. Y. (1978). Catecholamine innervation of the basal forebrain. Ii. Amygdala, suprarhinal cortex and entorhinal cortex. *J. Comp. Neurol.* 180 509–532. 10.1002/cne.901800308 659673

[B29] Fiorentino J. Payne M. Cancian E. Plonka A. Dumas L. Chirio D. (2022). Correlations between persistent olfactory and semantic memory disorders after SARS-CoV-2 infection. *Brain Sci.* 12:714. 10.3390/brainsci12060714 PMC9221020 35741601

[B30] Fletcher M. L. Wilson D. A. (2002). Experience modifies olfactory acuity: Acety)Lcholine-dependent learning decreases behavioral generalization between similar odorants. *J. Neurosci.* 22:Rc201. 10.1523/JNEUROSCI.22-02-j0005.2002 11784813 PMC2365514

[B31] Fyhn M. Molden S. Witter M. P. Moser E. I. Moser M. B. (2004). Spatial representation in the entorhinal cortex. *Science* 305 1258–1264. 15333832 10.1126/science.1099901

[B32] Glovaci I. Chapman C. A. (2015). Activation of phosphatidylinositol-linked dopamine receptors induces a facilitation of glutamate-mediated synaptic transmission in the lateral entorhinal cortex. *PLoS One* 10:e0131948. 10.1371/journal.pone.0131948 26133167 PMC4489908

[B33] Hafting T. Fyhn M. Molden S. Moser M. B. Moser E. I. (2005). Microstructure of a spatial map in the entorhinal cortex. *Nature* 436 801–806. 10.1038/nature03721 15965463

[B34] Halasz N. Johansson O. Hokfelt T. Ljungdahl A. Goldstein M. (1981). Immunohistochemical identification of two types of dopamine neuron in the rat olfactory bulb as seen by serial sectioning. *J. Neurocytol.* 10 251–259. 10.1007/BF01257970 6118395

[B35] Hansen K. A. Kay K. N. Gallant J. L. (2007). Topographic organization in and near human visual area V4. *J. Neurosci.* 27 11896–11911. 17978030 10.1523/JNEUROSCI.2991-07.2007 PMC6673353

[B36] Hargreaves E. L. Rao G. Lee I. Knierim J. J. (2005). Major dissociation between medial and lateral entorhinal input to dorsal hippocampus. *Science* 308 1792–1794. 10.1126/science.1110449 15961670

[B37] Hokfelt T. Ljungdahl A. Fuxe K. Johansson O. (1974). Dopamine nerve terminals in the rat limbic cortex: Aspects of the dopamine hypothesis of schizophrenia. *Science* 184 177–179. 10.1126/science.184.4133.177 4856104

[B38] Hoogland P. Welker E. Melzer P. Vanderloos H. (1987). Organization of the Projection from the barrel cortex to the thalamus in mice studied with the anterograde tracer phaseolus-leuco-agglutinin (Pha-L). *Acta Anatom.* 128 339–339.

[B39] Igarashi K. M. (2023). Entorhinal cortex dysfunction in Alzheimer’s disease. *Trends Neurosci.* 46 124–136. 36513524 10.1016/j.tins.2022.11.006 PMC9877178

[B40] Igarashi K. M. Ieki N. An M. Yamaguchi Y. Nagayama S. Kobayakawa K. (2012). Parallel mitral and tufted cell pathways route distinct odor information to different targets in the olfactory cortex. *J. Neurosci.* 32 7970–7985. 10.1523/JNEUROSCI.0154-12.2012 22674272 PMC3636718

[B41] Igarashi K. M. Lee J. Y. Jun H. (2022). Reconciling neuronal representations of schema, abstract task structure, and categorization under cognitive maps in the entorhinal-hippocampal-frontal circuits. *Curr. Opin. Neurobiol.* 77:102641. 10.1016/j.conb.2022.102641 36219950 PMC9818592

[B42] Igarashi K. M. Lu L. Colgin L. L. Moser M. B. Moser E. I. (2014). Coordination of entorhinal-hippocampal ensemble activity during associative learning. *Nature* 510 143–147. 10.1038/nature13162 24739966

[B43] Ikemoto S. (2007). Dopamine reward circuitry: Two projection systems from the ventral midbrain to the nucleus accumbens-olfactory tubercle complex. *Brain Res. Rev.* 56 27–78. 10.1016/j.brainresrev.2007.05.004 17574681 PMC2134972

[B44] Insausti R. Herrero M. T. Witter M. P. (1997). Entorhinal cortex of the rat: Cytoarchitectonic subdivisions and the origin and distribution of cortical efferents. *Hippocampus* 7 146–183. 10.1002/(SICI)1098-1063(1997)7:2<146::AID-HIPO4>3.0.CO;2-L 9136047

[B45] Kerr K. M. Agster K. L. Furtak S. C. Burwell R. D. (2007). Functional neuroanatomy of the parahippocampal region: The lateral and medial entorhinal areas. *Hippocampus* 17 697–708. 17607757 10.1002/hipo.20315

[B46] Koster N. L. Norman A. B. Richtand N. M. Nickell W. T. Puche A. C. Pixley S. K. (1999). Olfactory receptor neurons express D2 dopamine receptors. *J. Comp. Neurol.* 411 666–673. 10421875 10.1002/(sici)1096-9861(19990906)411:4<666::aid-cne10>3.0.co;2-s

[B47] Lechien J. R. Chiesa-Estomba C. M. De Siati D. R. Horoi M. Le Bon S. D. Rodriguez A. (2020). Olfactory and gustatory dysfunctions as a clinical presentation of mild-to-moderate forms of the coronavirus disease (Covid-19): A multicenter European study. *Eur. Arch. Oto Rhino Laryngol.* 277 2251–2261. 10.1007/s00405-020-05965-1 PMC7134551 32253535

[B48] Lee J. Y. Jun H. Soma S. Nakazono T. Shiraiwa K. Dasgupta A. (2021). Dopamine facilitates associative memory encoding in the entorhinal cortex. *Nature* 598 321–326. 10.1038/s41586-021-03948-8 34552245 PMC8744500

[B49] Leitner F. C. Melzer S. Lutcke H. Pinna R. Seeburg P. H. Helmchen F. (2016). Spatially segregated feedforward and feedback neurons support differential odor processing in the lateral entorhinal cortex. *Nat. Neurosci.* 19 935–944. 10.1038/nn.4303 27182817

[B50] Loureiro M. Achargui R. Flakowski J. Van Zessen R. Stefanelli T. Pascoli V. (2019). Social transmission of food safety depends on synaptic plasticity in the prefrontal cortex. *Science* 364:991. 10.1126/science.aaw5842 31171697

[B51] Luskin M. B. Price J. L. (1982). The distribution of axon collaterals from the olfactory-bulb and the nucleus of the horizontal limb of the diagonal band to the olfactory cortex, demonstrated by double retrograde labeling techniques. *J. Comp. Neurol.* 209 249–263. 10.1002/cne.902090304 7130455

[B52] Malach R. (1989). Patterns of connections in rat visual-cortex. *J. Neurosci.* 9 3741–3752. 2479724 10.1523/JNEUROSCI.09-11-03741.1989 PMC6569951

[B53] Miyamichi K. Amat F. Moussavi F. Wang C. Wickersham I. Wall N. R. (2011). Cortical representations of olfactory input by trans-synaptic tracing. *Nature* 472 191–196. 10.1038/nature09714 21179085 PMC3073090

[B54] Moberly A. H. Schreck M. Bhattarai J. P. Zweifel L. S. Luo W. Q. Ma M. H. (2018). Olfactory inputs modulate respiration-related rhythmic activity in the prefrontal cortex and freezing behavior. *Nat. Commun.* 9:1528. 10.1038/s41467-018-03988-1 29670106 PMC5906445

[B55] Mori K. Nagao H. Yoshihara Y. (1999). The olfactory bulb: Coding and processing of odor molecule information. *Science* 286 711–715. 10531048 10.1126/science.286.5440.711

[B56] Mori K. Takahashi Y. K. Igarashi K. M. Yamaguchi M. (2006). Maps of odorant molecular features in the mammalian olfactory bulb. *Physiol. Rev.* 86 409–433. 16601265 10.1152/physrev.00021.2005

[B57] Murata K. Kanno M. Ieki N. Mori K. Yamaguchi M. (2015). Mapping of learned odor-induced motivated behaviors in the mouse olfactory tubercle. *J. Neurosci.* 35 10581–10599. 10.1523/JNEUROSCI.0073-15.2015 26203152 PMC6605114

[B58] Nagayama S. Homma R. Imamura F. (2014). Neuronal organization of olfactory bulb circuits. *Front. Neural Circuits* 8:98. 10.3389/fncir.2014.00098 25232305 PMC4153298

[B59] Oettl L. L. Scheller M. Filosa C. Wieland S. Haag F. Loeb C. (2020). Phasic dopamine reinforces distinct striatal stimulus encoding in the olfactory tubercle driving dopaminergic reward prediction. *Nat. Commun.* 11:3460. 10.1038/s41467-020-17257-7 32651365 PMC7351739

[B60] Ohara S. Onodera M. Simonsen O. W. Yoshino R. Hioki H. Iijima T. (2018). Intrinsic projections of layer Vb neurons to layers Va, III, and II in the Lateral and medial entorhinal cortex of the rat. *Cell Rep.* 24 107–116. 10.1016/j.celrep.2018.06.014 29972772

[B61] Ojima H. Honda C. N. Jones E. G. (1991). Patterns of axon collateralization of identified supragranular pyramidal neurons in the cat auditory cortex. *Cereb. Cortex* 1 80–94. 10.1093/cercor/1.1.80 1822727

[B62] O’Keefe J. Dostrovsky J. (1971). The hippocampus as a spatial map. Preliminary evidence from unit activity in the freely-moving rat. *Brain Res.* 34 171–175. 10.1016/0006-8993(71)90358-1 5124915

[B63] Paxinos G. (2004). *The rat nervous system.* Amsterdam: Elsevier.

[B64] Petzold G. C. Hagiwara A. Murthy V. N. (2009). Serotonergic modulation of odor input to the mammalian olfactory bulb. *Nat. Neurosci.* 12 784–U142. 10.1038/nn.2335 19430472

[B65] Price J. L. (2003). Comparative aspects of amygdala connectivity. *Amygdala Brain Funct.* 985 50–58. 10.1111/j.1749-6632.2003.tb07070.x 12724147

[B66] Serby M. Larson P. Kalkstein D. (1991). The nature and course of olfactory deficits in Alzheimer’s disease. *Am. J. Psychiatry* 148 357–360. 1992839 10.1176/ajp.148.3.357

[B67] Sha M. F. R. Koga Y. Murata Y. Taniguchi M. Yamaguchi M. (2023). Learning-dependent structural plasticity of intracortical and sensory connections to functional domains of the olfactory tubercle. *Front. Neurosci.* 17:1247375. 10.3389/fnins.2023.1247375 37680965 PMC10480507

[B68] Shepherd G. M. (2004). *The synaptic organization of the brain.* Oxford: Oxford University Press.

[B69] Shiotani K. Tanisumi Y. Murata K. Hirokawa J. Sakurai Y. Manabe H. (2020). Tuning of olfactory cortex ventral tenia tecta neurons to distinct task elements of goal-directed behavior. *Elife*, 9. 10.7554/eLife.57268 PMC7423337 32749216

[B70] Stettler D. D. Axel R. (2009). Representations of odor in the piriform cortex. *Neuron* 63 854–864. 19778513 10.1016/j.neuron.2009.09.005

[B71] Sürmeli G. Marcu D. C. Mcclure C. Garden D. L. F. Pastoll H. Nolan M. F. (2016). Molecularly defined circuitry reveals input-output segregation in deep layers of the medial entorhinal cortex. *Neuron* 92 929–929. 10.1016/j.neuron.2016.11.011 PMC5652228 27883905

[B72] Tolman E. C. (1948). Cognitive maps in rats and men. *Psychol. Rev.* 55 189–208. 18870876 10.1037/h0061626

[B73] Tsao A. Moser M. B. Moser E. I. (2013). Traces of experience in the lateral entorhinal cortex. *Curr. Biol.* 23 399–405. 23434282 10.1016/j.cub.2013.01.036

[B74] Tse D. Langston R. F. Kakeyama M. Bethus I. Spooner P. A. Wood E. R. (2007). Schemas and memory consolidation. *Science* 316 76–82. 17412951 10.1126/science.1135935

[B75] Van Hoesen G. W. Hyman B. T. Damasio A. R. (1991). Entorhinal cortex pathology in Alzheimer’s disease. *Hippocampus* 1 1–8. 1669339 10.1002/hipo.450010102

[B76] Vandrey B. Armstrong J. Brown C. M. Garden D. L. F. Nolan M. F. (2022). Fan cells in lateral entorhinal cortex directly influence medial entorhinal cortex through synaptic connections in layer 1. *Elife* 11:e83008. 10.7554/eLife.83008 36562467 PMC9822265

[B77] Waldton S. (1974). Clinical observations of impaired cranial nerve function in senile dementia. *Acta Psychiatr. Scand.* 50 539–547. 4460686 10.1111/j.1600-0447.1974.tb09714.x

[B78] Wesson D. W. Wilson D. A. (2010). Smelling sounds: Olfactory-auditory sensory convergence in the olfactory tubercle. *J. Neurosci.* 30 3013–3021. 10.1523/JNEUROSCI.6003-09.2010 20181598 PMC2846283

[B79] Witter M. P. Wouterlood F. G. Naber P. A. Van Haeften T. (2000). Anatomical organization of the parahippocampal-hippocampal network. *Ann. N. Y. Acad. Sci.* 911 1–24. 10911864 10.1111/j.1749-6632.2000.tb06716.x

[B80] Young B. J. Otto T. Fox G. D. Eichenbaum H. (1997). Memory representation within the parahippocampal region. *J. Neurosci.* 17 5183–5195. 9185556 10.1523/JNEUROSCI.17-13-05183.1997 PMC6573311

[B81] Zaborszky L. Carlsen J. Brashear H. R. Heimer L. (1986). Cholinergic and GABAergic afferents to the olfactory bulb in the rat with special emphasis on the projection neurons in the nucleus of the horizontal limb of the diagonal band. *J. Comp. Neurol.* 243 488–509. 10.1002/cne.902430405 3512629

[B82] Zhang Z. Zhang H. Wen P. Zhu X. Wang L. Liu Q. (2017). Whole-brain mapping of the inputs and outputs of the medial part of the olfactory tubercle. *Front. Neural Circuits* 11:52. 10.3389/fncir.2017.00052 28804450 PMC5532451

